# Empowering Future Physicians: Transitioning from OSCEs to Smart Screen-Based Virtual Patients for Enhanced Clinical Competency Evaluation

**DOI:** 10.34172/aim.34736

**Published:** 2025-09-01

**Authors:** Zohrehsadat Mirmoghtadaie, Mohsen Keshavarz

**Affiliations:** ^1^Department of eLearning in Medical Sciences, School of Medical Education and Learning Technologies, Shahid Beheshti University of Medical Sciences, Tehran, Iran; ^2^Department of E-Learning in Medical Sciences, School of Paramedical Sciences, Torbat Heydariyeh University of Medical Sciences, Torbat Heydariyeh, Iran

## Introduction

 One of the primary challenges in contemporary medical education is creating assessments that evaluate essential clinical skills and reflect the diverse realities of patient care. In advancing healthcare systems and clinical competencies within the educational context, assessment methods have to adapt to multiple clinical contexts, demonstrate numerous scenarios and reflect the realities and complexities faced by clinicians assessing health care professionals. The influence of technology in medical education can be both advantageous and a hindrance.^1^ The use of smart screen based virtual patients can assist in creating tailored clinical scenarios that examine competencies holistically. These virtual settings present numerous clinical situations in which learners typically find themselves distracted, multi-tasking, managing time pressures, in a safe setting, and allows learners to practice the skills of diagnostic reasoning, making clinical decisions, and engaging with patients in a non-threatening environment.^2^ Transitioning from traditional observed structured clinical examinations (OSCEs) to virtual assessments creates uncertainty about the validity and reliability of the new education tools. The greater concern is that new educational assessment strategies are developed and use unique methodologies to assess the essential competencies health care professionals need to be credible and clinically competent. As medical education is developing competent, compassionate, and adaptable clinicians, the change to smart screen based virtual patients is an important transition in assessment methodologies. It is now imperative to comprehensively examine the effectiveness and educational value of these new smart screen-based processes to inform competency-based assessments, which will improve the quality of education, and in turn, improve patient care.

## What Is the Educational Challenge?

 The OSCE has been a fundamental tool in medical education, allowing educators to assess clinical skills with a standardized technique in a controlled clinical environment.^3^ However, with the realization that we are now in the digital age, there are a number of challenges that question the value of OSCEs in training the healthcare professionals of the future. Perhaps most importantly, OSCEs have not been able to address problems including lack of diversity in patient experiences, time-intensive organization, resource allocation, and the artificiality of the patient situation. OSCEs typically involve a limited number of standardized cases, and as a result, there is a mismatch in students’ education and preparation for diverse clinical contexts and cultural competencies. Although OSCEs provide important experiences, they require considerable time and resources in planning and execution, which are potentially problematic in terms of scalability in larger institutions. In addition, OSCEs might not help prepare students to manage the unpredictability of clinical contexts, because the experience is not typical of how students will function in their future roles. As medical education changes and evolves, it is important to prioritize underlying trends and obstacles in our approaches to training the healthcare professionals of the future, focusing on approaches and opportunities that leverage technology and enhance their training and assessment.

## What Are the Proposed Solutions?

 To address the inherent challenges of the traditional OSCEs, a viable option is smart screen-based virtual patients (SSBVPs).^2-4^ SSBVPs provide an immersive learning experience that also integrates artificial intelligence (AI) and natural language processing as part of the assessment of clinical competencies.

 SSBVPs can be utilized and interpreted using advanced software platforms to undertake the complexity of clinical contexts so that students can interact and engage with them through touch screen interfaces. SSBVPs allow dynamic interactions that mimic a conversation with a patient, and the various clinical situations present students with different contextual challenges that mitigate some of the challenges of OSCEs.^5^

 One enabled challenge of SSBPVs is that they can be modified in real time according to the student’s response to a scenario that mimics the unpredictability of clinical practice. The SSBVPs provide immediate, detailed feedback and assessments of performance on clinical reasoning and communication skills^5^, and SSBVPs used as a component of the curriculum offer blended learning that will advance skill development in a combination of controlled yet authentic learning.

## What Benefits Could Be Offered to a Larger Global Audience?

 The SSBVPs offer significant advantages from a clinical evaluation testing perspective, demonstrating their evolutionary development and potential for worldwide application as successful alternatives to traditional OSCE.

- Consistent and fair assessment: SSBVPs also offers a consistent assessment platform for evaluating clinical competencies, thereby increasing the impartiality and uniformity of assessments. This objectivity lowers bias and enhances the validity of the evaluation process all over the world.
- Scalability and flexibility: SSBVPs can accommodate many students at the same time with a single software platform as opposed to OSCEs while offering equivalent quality. SSBVPs allow students to engage with virtual patients when they choose and provide flexibility in scheduling evaluations that fit their specific learning needs.
- Realistic simulation of clinical scenarios: SSBVPs utilize the highest-end technological systems that underpin them enabling highly realistic interactions and simulations that are dynamic like real-world challenges. This provides a realistic experience for students who will eventually engage and interact with real patients to problem solve.
- Immediate feedback and growth opportunities: SSBVPs provide immediate feedback after students have engaged with their virtual patients enabling students to immediately view their strengths and weaknesses and affords higher growth opportunities since feedback is delivered in real-time. OSCE feedback is meaningful but oftentimes takes time to be delivered whether it is by classmates or by examiners.
- Variety and diversity of scenarios: SSBVPs provide a diverse range of clinical scenarios, exposing students to different types of challenges and prepare them for a globalized healthcare system in their daily practice.
- Cost-effectiveness: SSBVPs can be more cost-effective than the traditional OSCEs, thus allowing students to overcome logistical restrictions and decrease costs, making a comprehensive assessment option affordable to institutions that have no or limited budgets.

SSBVPs provide a new way of clinical assessment, using artificial intelligence and natural language processing to innovate medical training, preparing future medical practitioners to manage the complexities of modern-day practice.

## What Are the Next Steps?

 The addition of SSBPVs offers an exciting opportunity for enrichment of medical education and even the potential to supplant traditional OSCE. Education organizations can capitalize on the SSBVPs by coordinating and implementing the following strategies:

- SSBVP content creation: Develop a range of virtual patient cases that present clinical conditions and scenarios, patient demographics and cultural factors.
- Technology selection: Choose or develop appropriate, user-friendly software technology that leverages current technology used in educational institutions.
- Training and faculty preparation: Provide resources and workshops to assist educators in the implementation and assessment of SSBVP interactions.
- Curriculum integration: Use the curriculum objectives to drive alignment of the SSBVP assessments and use of the SSBVPs.
- Evaluation and feedback of implementation: Assess the effectiveness of the SSBVPs on various student outcomes and make adjustments to the design based on this feedback.
- Expanding use: Seek opportunities to expand the use of SSBVPs to other programs, and build collaborative practices with educational institutions.

With identified strategic actions, educational institutions can harness the potential of SSBVPs to enhance the education of future healthcare practitioners. In essence, Smart Screen-Based Virtual Patients offer a powerful and versatile tool for medical education, enabling learners to develop essential clinical skills in a safe, engaging, and cost-effective manner.
